# A Review of Current Methods for Analysis of Mycotoxins in Herbal Medicines

**DOI:** 10.3390/toxins10020065

**Published:** 2018-02-02

**Authors:** Lei Zhang, Xiao-Wen Dou, Cheng Zhang, Antonio F. Logrieco, Mei-Hua Yang

**Affiliations:** 1 Key Laboratory of Bioactive Substances and Resources Utilization of Chinese Herbal Medicine, Ministry of Education, Institute of Medicinal Plant Development, Chinese Academy of Medical Sciences & Peking Union Medical College, Beijing 100193, China; zhanglei-85@163.com (L.Z.); douxiaowen573@163.com (X.-W.D.); zcmycotoxin@163.com (C.Z.); 2 National Research Council of Italy, CNR-ISPA, Via G. Amendola, 122/O, I-70126 Bari, Italy

**Keywords:** herbal medicines, mycotoxin, sampling, sample pretreatment, chromatographic methods, rapid detection method

## Abstract

The presence of mycotoxins in herbal medicines is an established problem throughout the entire world. The sensitive and accurate analysis of mycotoxin in complicated matrices (e.g., herbs) typically involves challenging sample pretreatment procedures and an efficient detection instrument. However, although numerous reviews have been published regarding the occurrence of mycotoxins in herbal medicines, few of them provided a detailed summary of related analytical methods for mycotoxin determination. This review focuses on analytical techniques including sampling, extraction, cleanup, and detection for mycotoxin determination in herbal medicines established within the past ten years. Dedicated sections of this article address the significant developments in sample preparation, and highlight the importance of this procedure in the analytical technology. This review also summarizes conventional chromatographic techniques for mycotoxin qualification or quantitation, as well as recent studies regarding the development and application of screening assays such as enzyme-linked immunosorbent assays, lateral flow immunoassays, aptamer-based lateral flow assays, and cytometric bead arrays. The present work provides a good insight regarding the advanced research that has been done and closes with an indication of future demand for the emerging technologies.

## 1. Introduction

Herbal medicines, which are also referred to as phytomedicines or botanical medicines, have played a critical role in world health for thousands of years. According to the World Health Organization (WHO), “herbal medicines include herbs, herbal materials, herbal preparations and finished herbal products, that contain as active ingredients parts of plants, or other plant materials, or combinations” [1].

Over the last decade, the use of herbal medicines has expanded across the globe and gained considerable popularity. As a result of cultural and historical influences, herbal medicines remain an important part of the healthcare system in China, India, and Africa [2,3,4]. In recent years, the utilization of herbal medicines as complementary therapy has become more common in developed countries that have a typically well-established health care system structure [5,6]. According to the WHO, over 100 million Europeans currently use Traditional and Complementary Medicine (T&CM), and one-fifth among them regularly using T&CM for health care. It has been shown that there are many more T&CM users in Africa, Asia, Australia, and North America [7].

With increasing expansion in herbal medicine use globally, the quality control mechanisms surrounding the herbal medicines have become the main concern for both health authorities and the public. In the case of herbal medicines, contamination is critical to monitor, as toxicities related to extrinsic factors that are typically associated with undesirable toxic substances, rather than the herbs themselves, can result. In particular, fungal/microbial contamination has been a global concern for decades. According to prior investigations, toxigenic fungi species that are generated from soil or plants themselves can result in contamination of herbal medicines. These toxigenic fungi include species belonging to *Aspergillus*, *Penicillium*, *Fusarium*, and *Alternaria* genera [8,9,10]. Under unfavorable environmental conditions, these fungi produce mycotoxins, which are secondary metabolites that could contaminate various plants when in the field or at any stage during the collection, handling, transportation, or storage of the plants (e.g., mycotoxin contamination produced by *Fusarium* species can occur in the field and build up during the harvesting and drying stage, while additional toxins mainly produced by *Penicillium* and *Aspergillus* species can contaminate in storage operation) [11]. Reports regarding mycotoxin contamination screening of medicinal herbs and related products demonstrate that aflatoxins (AFs), ochratoxins, fumonisins (FBs), trichothecenes, and zearalenones (ZENs) are found to be the most commonly contaminated ones [12,13] (Table 1). These mycotoxins were identified to be carcinogenic, teratogenic, and mutagenic. In addition, they were also found to harm live cells, kidney, reproductive system, immune system, and central nervous system [14]. Among all the known mycotoxins, the most toxic one is aflatoxin B_1_ (AFB_1_). It was classified as a Group-1 carcinogen by the International Agency for Research on Cancer (IARC) due to its strong toxicity [15], and represents the main threat worldwide.

**Figure 1:**
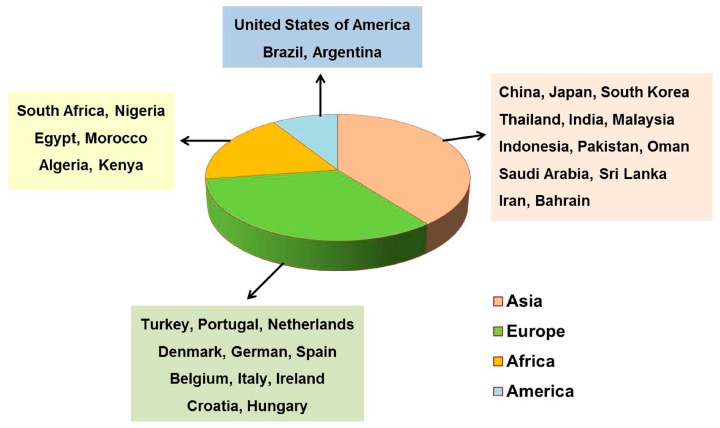
Distributions of representative countries that have published the reports of mycotoxins analysis in herbal medicine.

Currently, numerous published reviews reported the occurrence of mycotoxin contamination in herbal materials and related products. These reports indicated that mycotoxin contamination in herbal medicines is considered a global issue, particularly in the case of developing countries [16,33,34,35,36,37,38,39] (Figure 1). To date, more than 40 mycotoxins have been detected in herbal medicines [12,13,40]. The typical examples of these mycotoxins are shown in Table 1. In addition to the toxicity effects of mycotoxins themselves, the presence of mycotoxins in herbal medicines may also function to decrease the medicinal potency, lead to drug interactions, and potentiate adverse effects that could influence the safety of these herbal remedies [41].

Due to the hazardous effects associated with mycotoxins, approximately 100 countries implemented specific limits for the presence of mycotoxins in foodstuffs and feedstuffs by the end of 2003 [42]. National regulations have been established for numerous mycotoxins, including the naturally occurring AFs and aflatoxin M_1_ (AFM_1_), the trichothecenes deoxynivalenol (DON), diacetoxyscirpenol (DAS), T-2 toxin (T-2) and HT-2 toxin (HT-2), fumonisin B_1_, B_2_ and B_3_ (FB_1_, FB_2_ and FB_3_), agaric acid, ergot alkaloids (EA), ochratoxin A (OTA), patulin (PAT), phomopsins, sterigmatocystin (ST) and ZEN [42]. However, in the case of medicinal plants, official regulations regarding the presence of only AFs and OTA in medicinal herbs are shared globally in pharmacopoeias, national, and organizational regulations. In general, the current legal limit for AFB_1_ in herbal medicines ranges between 2 and 10 μg kg^−1^, while the limit for combined aflatoxin B_1_, G_1_, B_2_ and G_2_ (total AFs) ranges from 4 to 20 μg kg^−1^, and the limit for OTA ranges from 15 to 80 μg kg^−1^, as depicted in Table 2.

In regard to AFs, the European Union (EU) has set a limit of 5 μg kg^−1^ for AFB_1_ and 10 μg kg^−1^ for total AFs in nutmeg, ginger, turmeric, and pepper [43]. However, the European Pharmacopeia (EP) has implemented stricter limits for the presence of AF in herbal drugs, with the limits set to 2 μg kg^−1^ for AFB_1_ and 4 μg kg^−1^ for total AFs [44]. The same limit is set for the presence of AF in herbal drugs, which was set by the British Pharmacopeia (BP) [45]. Germany has implemented a limit of 2 μg kg^−1^ for AFB_1_ and 4 μg kg^−1^ for total AFs in any materials that are used in manufacturing of medicinal products (including medicinal herbal products) [46]. In the USA, a limit of 5 μg kg^−1^ has been implemented for AFB_1_ and 20 μg kg^−1^ for total AFs was established by the United States Pharmacopeia (USP) for certain types of raw medicinal herb materials, as well as their powder and/or dry extract [47,48]. Identical limits have been set by Argentina for herbs, herbal materials, and herbal preparations that are used in herbal tea infusions [46]. In addition, Canada has implemented the same legislation regarding products that contain ginseng or any substance derived from this source, including evening primrose oil, sugar cane, sugar beets, and cottonseed [49]. In China, a total of nineteen different types of traditional Chinese medicines (TCMs) medicinal herbs are regulated in order to detect AF, with the limits being 5 μg kg^−1^ for AFB_1_ and 10 μg kg^−1^ for total AFs. In order to regulate AF levels [50], South Korea has also established limits of 10 μg kg^−1^ for AFB_1_ and 15 μg kg^−1^ for total AFs in sixteen types of medicinal herbs [51]. Japan has set a limit of 10 μg kg^−1^ for total AFs in crude drugs as well as preparations containing crude drugs as the primary ingredient (crude drug preparations) [52]. Indonesia has set a legislative limit of 20 μg kg^−1^ for total AFs in the category of “coconut, spices and traditional drug medicines/herbs” [42]. In Vietnam, limits of 5 μg kg^−1^ for AFB_1_ and 10 μg kg^−1^ for total AFs have been implemented for dry white and black pepper, nutmeg, ginger, and turmeric [53]. Compared with AF, only few countries or organizations, such as Vietnam [53] and the EU [43], have established a maximum residue level (MRL) for OTA in nutmeg, ginger, turmeric, black and white pepper, liquorice root and its extract, with the legislative limit varying from 15 μg kg^−1^ to 80 μg kg^−1^.

In order to satisfy the requirements of the recent legislation and to protect consumer health, it is imperative that sensitive methods be developed for mycotoxin analysis. The development of accurate and rapid methods for the determination of mycotoxin levels in herbal medicines remains a challenging task due to the trace level of mycotoxin, as well as the involvement of an extremely complicated matrix. Therefore, in contrast to the analytical technology that is utilized in general food and feed, methods for medicinal herbs typically require modification along with characters of different types of matrixes, which are primarily embodied in the sample preparation. Numerous reviews have focused on the occurrence of mycotoxins in herbal medicine [16,33,54,55,56,57]; however few provided detailed summaries of the development of the analytical methods utilized for mycotoxin determination. A previous review reported by our group in 2012 focused on the development of mycotoxin detection methods in TCMs [58]. In recent years, the application of biotechnology and nanotechnology has greatly pushed the analytical techniques forward. Here, this review thoroughly summarizes the advances and progress of the analytical methods from sampling, pretreatment to detection of mycotoxin contamination in herbal medicines. In addition, we review the recent development of screening assays used for mycotoxin detection in herbal medicines.

## 2. Sampling, Extraction and Cleanup

### 2.1. Sampling

Sampling plays a critical role in how precise the determination of mycotoxin levels are due to the fact that the molds that generate mycotoxins do not grow uniformly on the substrate and existing contamination in natural samples is not homogeneous. A study carried out in 2003 demonstrated that the actual mycotoxin concentration of a bulk lot cannot be determined with 100% certainty due to the variability associated with each step in the mycotoxin test procedure. Thus, the sampling procedure could dramatically impact the final results regarding the determination of mycotoxins [59].

In order to standardize the sampling procedure for mycotoxin testing, Commission Regulation (EC) No 401/2006 was set in order to lay down the sampling methods and analysis for the official control of mycotoxin levels in foodstuffs [60]. This was revised in 2010 and 2014, respectively [61,62]. For example, with spices, the incremental samples should be taken depending on the weight of the lot. In the case of lots that weigh equal to or greater than 15 tons, 100 incremental samples should be taken from sub lots that make up a 10 kg aggregate sample weight. In the case of samples weighing less than 15 tons, 5 to 100 incremental samples should be taken depending on the lot weight, resulting in an aggregate sample weight of 0.5 to 10 kg. It should be noted that the earlier legislation Commission Directive 2002/27/EC also regulates the sampling methods utilized for AF analysis in spices. Certain distinctions exist between these two regulations. Specifically, according to Directive 2002/27/EC, when the weight of the lot is less than 15 tons, 10 to 100 incremental samples should be taken that make up a 1 to 10 kg aggregate sample weight [63].

In addition, the method for sampling bulk and retail herbal material packages has been included in the guidelines published by the WHO in regard to quality control methods for herbal materials [64]. In terms of sampling from bulk material, when a batch consists of five containers or packaging units, a sample must be taken from each. In addition, it is also recommended that in the case of batches with 6–50 units, samples from five should be taken. In the case of batches including greater than 50 units, samples must be taken from 10% of the individual units, and the number of units must be rounded up to the nearest multiple of 10. In regard to sampling material from retail packages, when each wholesale container (box, carton, etc.) is selected for sampling, two consumer packages must be taken at random. In the case of small batches (1–5 boxes), a total of 10 consumer packages should be taken.

In some instances, the sampling plan was carried according to particular experience. For example, when Philip Müller et al. studied AF contamination of Indian *Cassia senna* L. (Caesalpinaceae) pods prior to harvest, during the drying procedure, and during storage, they found it necessary to take a minimum sample size of 2 kg of the material randomly in order to obtain a representative sample. In the case of instances when there was greater than 400 kg of the total stock material, over 500 g/100 kg of pods were selected for analysis [65].

In summary, of the studies published regarding mycotoxin analysis in herbal medicine, the majority of the samples were randomly collected from two sale terminals (public markets and drugstore), while some studies reported the collection of samples from herbal medicine users [66]. In the majority of these reports, a very small quantity of the lot was used in the end for contamination quantification. However, only few studies provided a detailed description of the sampling procedure used [67,68]. The sampling step typically represents the largest source of error due to the extreme distribution of mycotoxins among kernels within the lot [59]. Therefore, a reasonable sampling plan will help to minimize the risk of misclassifying the product, which could further facilitate trade as well as provide consumer protection. Thus, it is suggested that researchers pay more attention to the sampling procedure in the future studies.

### 2.2. Extraction Procedure

The purpose of extraction is to remove mycotoxin from the herbal medicine matrix as much as possible into a solvent that is suitable for subsequent cleanup or direct analysis. The extraction solvent and method used are the two most important considerations for the extraction procedure.

#### 2.1.1. Extraction Solution

The selection of the extraction solvent depends on several things, including physical and chemical characteristics of the analyte, solvent cost and safety, the solubility of the non-analyte in the extraction solvent and subsequent processing steps following extraction. Ideally, the extraction solvent should remove only the mycotoxin of interest from the sample matrix. However, due to the complex matrix of herbal medicines and the absence of a completely specific extraction solvent, the extraction solvent used should be adjusted according to the characteristics of both the analyte and associated matrix. 

Currently, the most common solvents used for the extraction of mycotoxins from herbal medicines are methanol-water and acetonitrile-water (Table 3 and Table 4). However, in order to enable higher extraction efficiencies and lower matrix effects (MEs), the extraction solvents still need to be compared across many studies. The improved efficiency of acetonitrile-based solvents compared to methanol has been demonstrated by some groups. It was demonstrated not only for the determination of single type of mycotoxin present in TCMs, such as FBs [69], but also for ZEN and its related mycotoxins [70], DON, Nivalenol (NIV) [71], and the simultaneous detection of multiple mycotoxins [13].

In addition to of the commonly used solvents (e.g., methanol and acetonitrile), other solvent types such as ethanol [72], acetone [73], ethyl acetate [12,74] and chloroform [75,76] are also used sometimes for mycotoxin extraction from herbal medicines. It should be noted that if ethyl acetate is used as the extraction solution, an extra defatting procedure may be required prior to cleanup or detection due to the high levels of fatty matrix compounds that are known to be co-extracted with the ethyl acetate-containing solvent [12].

There could be clear differences in the recovery as a result of varying the percentage of organic solvent. Wang and co-workers carried out a study to investigate the effect of five different methanol/water ratios (75%, 80%, 85%, 90% and 100% methanol) on the simultaneous extraction of AFB_1_ and OTA from licorice roots and fritillary bulbs. This study demonstrated that the highest extraction efficiency was obtained using a methanol/water ratio of 85% [77]. Another group reported a method for simultaneous determination of seventeen mycotoxins in Puerariae lobatae radix. In this study, acetonitrile/water (90:10, *v*/*v*) was selected as the extraction solvent after comparing the extraction efficiency of three different ratios (80%, 90% and 100%) of an acetonitrile/water solvent system [78].

The necessity to compare the proportion of organic solvent was more systematically demonstrated by a recent report regarding the analysis of AFs in TCMs. In this study, the matrix was divided into several types (volatile oils, proteins, polysaccharides and fatty oils), and five different ratios of aqueous methanol solutions were evaluated as the extraction solvent for each type. These studies demonstrated that a 75% aqueous methanol solution was the optimal solvent for volatile oils, while a ratio of 85% was optimal for proteins and 70% for polysaccharides and fatty oils [79].

A large portion of methanol or acetonitrile is used as an extraction solvent; however, a low portion of organic solvent is sometimes helpful in order to obtain satisfactory results. For example, when type A trichothecenes in coix seed were determined in the work of Dong et al., it was found that the percentage of acetonitrile used in the extraction solvent was critical. Specifically, the recoveries were found to increase along with decreasing acetonitrile percentage. When 2% acetonitrile was applied, satisfactory recoveries were obtained for all mycotoxins analyzed [135]. This could be explained by the fact that the material involved in the subsequent procedure for cleanup was easy to disperse into the aqueous solution, leading to an efficient purification.

Additional reagents were sometimes required to assist in the extraction. For example, acid (e.g., formic acid, acetic acid) and salt were required for the analysis of mycotoxins. Some studies have implied that the addition of proper ratio of formic acid to the extraction solvent could improve recoveries of AFs and FBs [139,141,148]. Higher recoveries and lower MEs were obtained with the addition of 1% acetic acid to the extraction solvent when 11 mycotoxins were simultaneously determined in malt [144]. The addition of proper NaHCO_3_ into the extraction solution could function to improve the recoveries of AFs and OTA in ginseng and ginger matrices [80].

The addition of water typically improves the extraction efficiency due to the fact that water increases penetration of the solvent into the material by breaking interactions between toxins and other sample constituents, such as proteins or sugars [149]. However, it should be noted that solvent including water was not tolerated in the case of some matrices. In a study carried out by Chen et al. [138], the extraction solvent was optimized for the determination of 10 mycotoxin contaminants in *Panax notoginseng*. This study demonstrated that water would make a lot of saponins dissolve, which significantly affected the detection signal. Therefore, it was found that a 100% acetonitrile solution was the optimal solvent.

Often, if the efficiency of the one step extraction is satisfactory, it is not necessary to repeat the extraction. Otherwise, two types of solvents can be used successively to carry out a two-step extraction method in order to obtain increased extraction efficiency [140,142]. In addition, extra management is required prior to extraction in the case of the detection of certain mycotoxins in herbal medicines. For example, PAT is prone to combine with a protein that originates from herbs to generate the complexity of PAT. Therefore, in the case of PAT determination in Fructus Crataegi and Fructus Mume, samples must be pretreated with pectinase in order to dissociate PAT from protein and obtain the dissociated PAT molecule [132].

#### 2.2.2. Extraction Method

In addition to the type of extraction solvent used, the extraction method is another critical determinant of the extraction efficiency. The conventional solid-liquid extraction technology used for mycotoxin extraction involves the use of ultrasonic extraction, homogenization, and shaking. Vortexing and blending are also used sometimes for the detection of mycotoxins in herbal medicines. When selecting the extraction method, the matrix constitution should be considered. A recent report demonstrated that samples with different matrix types required their own specific extraction method. For example, in the case of matrices with high fatty oil and polysaccharide contents that are more viscous, an ultrasonography extraction method was found to be prone to aggregating the extracts and thereby prevented the dissolution of AFs from the matrices [79].

The extraction time required, as well as the number of samples analyzed, is also important considerations. Homogenization represents the most rapid method in comparison to other methods, with an extraction time of only 1–5 min [94,104,129]. However, homogenization is not applicable for the simultaneous extraction of numerous samples. Therefore, when large numbers of samples must be processed, an ultrasonic extraction method, which is easy to carry out, represents a good choice, while shaking requires a longer extraction time [32,79,128].

In addition to the extraction format used, the extraction time must also be optimized in order to obtain increased extraction efficiencies [79,146]. It should be noted that a too long extraction period could result in increased MEs as the starch disperses or forms glue, reducing mycotoxin recovery [150]. In addition, the extraction temperature could also impact extraction recovery. Extraction recovery has been demonstrated to typically increase with increasing temperature. However, in order to avoid co-extracting a large fraction of interferes, and to prevent the degradation of unstable mycotoxins, it is proposed to ensure that an extraction temperature of greater than 40 °C not be used [146].

It should be noted that extraction is preceded by a maceration step, which is helpful to obtain the highest possible extraction efficiency. The addition of water to wet the sample enables the release of analytes bound to the matrix [151]. In addition, the maceration procedure allows water to dissolve and remove water-soluble substances that may form a barrier to prevent extraction solvent from reaching the herb. This step therefore increases the availability of analytes for extraction by the followed extraction step [152].

As reported by Zhang et al. [148], mycotoxin recoveries were demonstrated to increase with an increasing soaking time, with a 30 min soaking time found to be adequate. The maceration step has also been utilized in other studies when mycotoxins were identified in herbal medicines. For example, the extraction recoveries were found to be significantly improved when the maceration step was carried out for 20 min followed by a 70 min extraction step [128].

The primary drawback associated with the extraction techniques mentioned above is that they are solvent and labor intensive. As the number of samples constantly increases, there is an increased interest in the development of more rapid and automatic approaches for sample extraction. Accelerated solvent extraction (ASE) is currently one of the most promising isolation procedures using organic solvents at a high pressure and at temperature above the boiling point [153]. High extraction efficiencies were obtained by varying significant factors, including extraction pressure, temperature, time, and the number of cycles. Recently, ASE was applied for the co-extraction of multiple mycotoxins from different TCMs by Han et al. [13]. This technique was compared to traditional extraction techniques, including ultrasonic and homogenization methods. The studies demonstrated that the optimal ASE method exhibited higher extraction efficiency, allowing for complete extraction using a minimum amount of solvent in a short period of time. This study demonstrated that high temperatures generally increase the extraction rate by both improving the solubility of the analytes and decreasing the viscosity and surface tension of the extraction solution. However, it should be noted that higher temperatures may cause degradation of the analytes or cause the analytes to react with the matrix. Supercritical fluid extraction (SFE) represents another alternative to the solvent-intensive extraction procedures, and has gained increased attention in the field in regards to removing influences caused by the matrices. Studies have demonstrated that analytes can be extracted by changing the pressure and temperature. One of the most important advantages of SFE over conventional extraction techniques is the pre-concentration effect, an effect crucial for trace analysis. Studies have demonstrated that mycotoxins could be extracted by SFE in TCMs. Specifically, Liu et al. [121] demonstrated good recovery results when SFE was used to extract AFs from Zizyphi Fructus. In this study, the developed SFE procedure was shown to efficiently eliminate matrix interferences by the removal of the majority of polar substances.

### 2.3. Cleanup

Considering the low residue level of mycotoxins (generally at μg kg^−1^ level) and the complex chemical composition of herbal medicine samples, a cleanup step was required prior to instrumental analysis in most cases (Table 3 and Table 4). This cleanup step may function to further concentrate mycotoxins in addition to removing sample impurities. A variety of cleanup methods have been implemented and shown to contribute to the accurate measurement of mycotoxins in herbal medicine, including solid phase extraction (SPE) and immunoaffinity column (IAC).

#### 2.3.1. SPE

##### Conventional SPE

SPE columns with various commercially available packing have been utilized for mycotoxin cleanup [149,154]. For example, SPE cartridges bonding C-18 sorbent were used to purify AFs from herbal samples. These are rich in fatty oils in order to protect the columns from damage in the subsequent test procedure [79], or to perform simultaneous cleanup for AFB_1_ and OTA in licorice roots and fritillary bulbs [77]. In addition, a strong anionic exchange (SAX) column was used for the purification of FBs from African traditional herbal medicines [101], as well as certain herbs and spices [133].

Satisfactory results were usually obtained using only one type of SPE column for cleanup. However, in the case of multimycotoxins with various polarities, as well as in the case of complicated matrices, two types of SPE columns have been proposed to be used in combination. For example, when multimycotoxins were analyzed in raw tea and herbal infusion materials, a NH_2_-SPE column and C_18_-SPE column were proposed to be used in combination in order to recover all of the described mycotoxins [74]. In detail, samples were extracted using ethyl acetate/formic acid (99:1, *v*/*v*). The extract was divided into two parts; one part was cleaned up using an NH_2_-SPE column followed by a C_18_-SPE column; another part was passed through the same C_18_-SPE column. Finally, the two elutes were combined for analysis. As another example, Ferreira et al. used a silica cartridge followed by a C_18_ cartridge in order to purify AFs in pepper [75].

##### Special SPE

Commercially available columns exist for a single type of mycotoxin (AFs, DON, FBs, etc.). In addition, multifunctional columns are available for the simultaneous determination of different groups of mycotoxins. For example, a TC-M160 column was used for the purification step of AFs in Pu-erh tea [111]. Yue et al. proposed the use of Puri Tox^SR^ TC-T200 DON as a cleanup step for the simultaneous determination of DON and NIV in TCMs [71]. AFs and PAT in Chinese patent medicines were simultaneously purified using a mycosep 228 Aflapat multifunctional column [130]. MultiSep 211 Fum columns were used to analyze FBs in TCMs [69]. Mavungu et al. carried out the cleanup procedure using Oasis HLB^TM^ SPE cartridges to identify 23 mycotoxins in botanical supplements [12]. Chen et al. purified 10 mycotoxins from *Panax notoginseng* using a HLB multifunction cleanup column [138]. A TC-M160 column was utilized to purify 17 mycotoxins from Puerariae lobatae radix [78].

##### Home-Made Cartridge

Because of the diversity of herbal medicine matrices, satisfactory results are not always obtained when using commercial SPE columns. Therefore, homemade cartridges have been proposed for mycotoxin cleanup steps in some cases. There are two critical points to bear in mind in regard to making homemade cartridges, including lower adsorbents of analytes and higher adsorbents of herbal medicine matrices, such as pigments. Currently, silica gel, alumina, and kieselguhr are three adsorbent materials that are commonly used for mycotoxin cleanup in herbal medicine matrices. Wu’s group has published a series of studies regarding the use of homemade cleanup cartridges for the determination of mycotoxins in TCMs. For example, the silica gel was used for purification of 35 mycotoxins [13], while the mixture of silica gel and alumina was used to purify AFB_1_, AFB_2_, AFG_1_, AFG_2_, AFM_1_ and AFM_2_ [129]. In addition, cartridges filled with equal proportions of alumina base, florisil, and kieselguhr have been used for cleanup of ZEN, α-Zearalenol (α-ZOL), β-Zearalenol (β-ZOL), Zearalanone (ZAN), α-Zearalanol (α-ZAL), and β-Zearalanol (β-ZAL) [70]. 

##### New Absorbents

Despite the conventional types of absorbent available, currently, some advanced nanomaterials have been used for mycotoxin determination, including carbon nanomaterial and magnetic carbon nanomaterial. The primary advantage of carbon nanomaterials is their high adsorption capacities due to their unique electronic, mechanical, and chemical properties [155]. Graphene oxide (GO) is the oxidized derivative of graphene, which is a type of representative carbon nanomaterial. GO is rich in oxygen atoms on the surface, including epoxy, hydroxyl, and carboxyl groups. These groups play a critical role in the formation of hydrogen bonds or electrostatic interactions with organic compounds containing oxygen- or nitrogen-functional groups. In addition, GO is able to adsorb aromatic rings from certain organic compounds through strong π-π interactions [155]. Recently, GO was used for the first time in the pre-concentration step in the extraction of AFs from traditional proprietary Chinese medicines [103]. The stacking between the benzene rings of AFs and GO, in addition to the hydrogen bonds formed between the oxygen containing groups contained in AFs and GO could be responsible for the adsorption of AFs on GO absorbent. However, in this report, a single pretreatment by GO was unable to meet the requirement of sensitive detection. In an effort to remove as much interference as possible, a cleanup procedure involving an MgSO_4_/NaCl salt mixture was carried out prior to the GO pre-concentration step.

Multi-walled carbon nanotubes (MWCNTs) are another type of carbon nanomaterial that is comprised of several rolled-up graphite sheets. MWCNTs have been demonstrated to adsorb type A trichothecenes and therefore, were used as SPE sorbents for the purification and enrichment of mycotoxins in maize, wheat, and rice [156]. In recent studies carried out by Han’s group [135,147], MWCNTs were incorporated with magnetic material to form magnetic-SPE adsorbents. These could be collected using an external magnetic field and recycled with a simple washing step, thereby achieving a rapid and easy protocol. First, magnetic-SPE adsorbents were successfully applied to purify four type A trichothecenes (T-2, HT-2, DAS and Neosolaniol (NEO)) in coix seed, and were subsequently used for the simultaneous enrichment and purification of ZEN and four type A trichothecenes in *Salviae Miltiorrhizae* Radix et Rhizoma.

While useful for the analysis of mycotoxins, the nanomaterials used in the studies discussed above were all self-synthetized, which could limit the scope of the application of these materials. In addition, only AFs, ZEN, and the four type A trichothecenes (T-2, HT-2, DAS and NEO) were investigated. The appropriate nanomaterials are awaiting evaluation for numerous other types of mycotoxins.

SPE has been demonstrated to be a safe, efficient, and reproducible technique. However, because herbal medicines are rich in secondary metabolites, including pigments, flavone, essential oils, polysaccharide, and fatty acids that could interfere with mycotoxin analysis, even if the samples are purified by SPE extraction, in most cases it still requires highly sensitive and selective detectors, such as mass detector to meet the requirements (Table 4). Therefore, cleanup methods with higher specificity are necessary.

#### 2.3.2. IAC

IAC, a method based on the interaction between antigen and antibody, exhibits some merits, including a minimal loss of mycotoxins and a maximal elimination of interfering substances. Therefore, compared to SPE extraction, the utilization of IAC as a cleanup procedure could greatly improve the specificity of subsequent analysis, thereby lowering the requirements of the detector.

For the determination of AFs in herbal medicine, IAC is most frequently used and is an efficient cleanup method that has been recommended by numerous related official organizations [44,47,50,51,52]. However, antibodies are prone to the influence of the herbal matrix, and the commonly used IAC approach therefore typically yields low recoveries [85,87,105,107]. In order to improve the recoveries of AFs in herbal matrix, numerous studies have been carried out in an effort to analyze potential factors that could be responsible for low rates of AF recovery. These studies have proposed solutions to mitigate these factors. For example, in the case of AFs, it was mentioned in the study of Ip et al. [100] that high extract acidity could lead to low recovery of AFs from certain medicinal herbs, especially in the case of AFG_2_. It was suggested that this problem can be reconciled through the use of 0.1 M phosphate buffer as the dilution solvent, as it has a higher buffering capacity compared to PBS and does not contain sodium chloride. Some studies have implied that a higher recovery of AFG_2_ was achieved when a more diluted sample extract was applied to the IAC [102,107]. Moreover, other studies [86,87] have demonstrated that addition of appropriate concentration of Tween-20 could function to improve AF recovery. However, these solutions were aimed at only a single type of matrix, with little attention focused on extending the applicability of other types of medicinal herbs. Recently, Yang’s group [94] carried out a systematic study to investigate critical points during the IAC cleanup procedure that are crucial for the detection of AFs in medicinal herbs. This group proposed a practical strategy that would be widely applicable to different types of herbal materials. Specifically, they focused on three impacts: (1) eliminating the effect of high acidity, (2) reducing the appearance of precipitate and nonspecific adsorption, and (3) decreasing the ME. These results demonstrated that a satisfactory recovery could be obtained when the sample extract was diluted in 0.1 M phosphate buffer solution (pH 7.8, 2% Tween-20) at a 1:8 dilution ratio.

In addition to AFs, there have been reports of using IAC to purify other types of mycotoxins from herbal medicines, including OTA [114], ZEN [119,134], and citrinin (CIT) [117]. In addition, certain IACs that can be used to clean up multi-mycotoxins have been proposed for the purification of mycotoxins from herbal medicines, such as AflaOchra Test^TM^ immunoaffinity column (VICAM, Milford, MA, USA) [86,87,127], and AflaZearal Test^TM^ immunoaffinity column (VICAM, Milford, MA, USA) [89]. 

The development of IAC has greatly improved the specificity of the cleanup method. However, the antibodies used in IAC sorbent have some down sides, including expensive cost, cross-reactivity, and poor tolerance to organic solvents.

#### 2.3.3. Aptamer-Affinity Column (AAC)

Aptamers are single-stranded (ss) oligonucleotides that are capable of recognizing target molecules with high affinity and specificity, similar to the properties of antibodies [157]. Compared with antibodies, aptamers offer significant advantages, including a lower cost and less labor intensive. In addition, aptamers that are immobilized on solid phases can be recycled, as they are easily regenerated within several minutes at room temperature [158,159]. Therefore, aptamers represent a promising tool for use in mycotoxin cleanup steps from complex matrices.

In 2013, Yang et al. [116] first prepared an OTA AAC using a covalent immobilization strategy. Next, AAC was successfully used to absorb OTA in ginger powder. In this study, both accuracy and reusability were compared between AAC and IAC. On one hand, it was found that there was a greater number of interfering peaks following cleanup with AAC compared to with IAC. However, on the other hand, no significant differences were found between the recoveries from these two cleanup procedures. Furthermore, cleanup with AAC was found to be less time-consuming. In addition, it was found that while AAC could be reused eight times without notable effects on aptamer-binding efficiency in ginger powder samples, IAC could only be reused four times. It should also be noted that it took only a few minutes to regenerate AAC for reusing, while it took >12 h for IAC to be regenerated for reuse.

Subsequently, this study [131] thoroughly validated the applicability of AAC for use with various types of TCM matrices, including fruits, seeds, rhizomes, roots, flowers, grasses, leaves, and animals. Satisfactory recoveries and enrichment purification effects were obtained using the AAC-based cleanup method, and this result further indicated that AAC possesses a promising application prospect in trace analysis.

#### 2.3.4. Molecularly Imprinted Polymers (MIPs)

MIPs are synthetic polymers that are capable of highly specifically recognizing of target analytes. The analyte retention results from a shape recognition in artificial binding sites that recognize the target molecule. In the most commonly used preparation process, monomers form a complex with a template through covalent or non-covalent interactions, and are then joined using a cross-linking agent [160]. MIPs exhibit clear advantages over true antibodies, including a high binding capacity, stability in extreme environments, and a relatively low synthesis cost [161]. In addition, MIPs provide promising advantages for small molecular weight mycotoxins, for which selective antibody development has been demonstrated to be difficult [162]. However, as summarized by Pereira et al. [149], certain mycotoxins are too toxic or too expensive to be used in an MIP preparation and can pose a number of problems. These problems include inconsistent molecular recognition, polymer swelling in unfavorable solvents, slow binding kinetics, and potential sample contamination by template bleeding.

Molecularly imprinted solid-phase extraction (MISPE) devices for mycotoxin detection have become commercially available. Cao et al. [115] has demonstrated the use of MIP-based SPE columns for a cleanup protocol for OTA in ginger. This study demonstrated that MIP exhibited a similar recovery compared to IAC. In addition, following a simple regenerated procedure, the MIP-based SPE column exhibited excellent stability, and could be reused at least forty-one times and obtain greater than 80% OTA recovery rates with ginger samples.

#### 2.3.5. QuEChERS

Quick, Easy, Cheap, Effective, Rugged, and Safe (QuEChERS) is a pretreatment technology that was originally developed in 2003 for pesticide determination [163]. This technology includes an extraction/partitioning step using acetonitrile and salts followed by a cleanup step that is based on a dispersive solid-phase extraction (dSPE) [164]. Due to its simplicity to operate, QuEChERS-based approaches have been used increasingly within the field for the extraction and purification of multi-mycotoxins from herbal medicine matrices.

During the extraction step, acetonitrile is primarily used as the solvent. However, it has also been proposed to soak the dry samples in water or PBS/NaH_2_PO_4_ buffer prior to the addition of extraction solvent, a step that is advantageous for later extraction [137,140,148]. In addition, because certain mycotoxins are pH-sensitive, such as FBs, OTA, and Ochratoxin B (OTB), proper formic acid is typically added to the acetonitrile in order to generate a low pH to prevent generation of the ionized form, which could contribute to satisfactory recoveries. For example, in the study that developed a method for the simultaneous determination of 22 mycotoxins in *Pheretima*, different ratios of formic acid in acetonitrile were investigated. This study demonstrated that recoveries of FBs, OTA, and OTB were less than 10% when using 1% formic acid. While when the percentage of formic acid was increased to 10%, satisfactory recoveries of OTA and OTB were obtained. However, FBs recoveries were only at 40–70%. It was determined that when 15% formic acid was used, FBs recoveries were greater than 80%. This could be due to the fact that FBs contain more carboxylic acid groups relative to OTA and OTB, which requires a lower pH in order to maintain their molecular form (a more extractable form) [148].

In regard to the phase separation steps, magnesium sulfate and sodium chloride are typically used in order to reduce water in the sample. This is sometimes added along with anhydrous trisodium citrate and sodium citrate dibasic sesquihydrate due to the fact that the citrate system has an amortizing role, making pH-sensitive mycotoxins, such as FBs, acquire satisfactory recoveries [137,140,142].

In regard to the subsequent cleanup procedure of QuEChERS, C_18_, primary secondary amine (PSA) and graphitized carbon black (GCB) are the typically used sorbents. It should be noted that PSA is prone to absorption of acidic mycotoxins such as FB_1_ and FB_2_, while the GCB adsorbent is prone to adsorption of mycotoxins that possess a planar structure, such as AFB_1_, AFB_2_, AFG_1_, AFG_2_, and ST [142].

In some cases, the cleanup step based on dSPE is removed or replaced by another purification protocol. For example, in the study of Liu et al. [140], a QuEChERS-based extraction protocol was applied for the simultaneous analysis of 8 different mycotoxins, including AFB_1_, AFB_2_, AFG_1_, AFG_2_, OTA, FB_1_, FB_2,_ and ZEN. The recoveries of these analytes were obtained from 78.9–97.8% only when an extraction/partitioning step was carried out. In 2013, Arroyo-Manzanares et al. [137] proposed a method for the determination of 15 mycotoxins in milk thistle using ultra high performance liquid chromatography-tandem mass spectrometry, which was used to determine FB_1_, FB_2_, NIV, DON, and fusarenon-X (FUS-X) following sample treatment with a modified method using a QuEChERS-based protocol. A subsequent cleanup step based on dispersive liquid-liquid microextraction was used to determine the remainder of the mycotoxins. Using a cleanup step with a commercial kit was found to result in decreased recoveries of all mycotoxins.

#### 2.3.6. One-Step Extraction

In general, an extra purification procedure is required for sample preparation of herbal medicines. However, in the case of some relatively simple matrices, it has been shown that it is not necessary to carry out this further cleanup procedure following extraction [32,139,141,143,144]. This type of protocol is considered to be a one-step extraction method. The majority of studies used a further concentration step to improve the sensitivity, or a dilution step to lower MEs. For example, when the method for the determination of multi-class mycotoxins in Chinese yam was developed, the sample (1.0 g) was extracted using 4 mL methanol-water-formic acid (79:20:1, *v*/*v*/*v*) with a 20 min ultrasonication treatment. A total of 1 mL of the extracted material was dried using nitrogen gas and re-dissolved in 0.5 mL methanol-water (50:50, *v*/*v*) for analysis [141]. A similar simple pre-treatment protocol was also used in studies carried out by Liu et al. [139,143]. However, in these studies, a one-fold dilution was used after extraction in place of the concentration step.

## 3. Analytical Techniques of Mycotoxins

### 3.1. Chromatographic Techniques for Detecting/Quantifying Mycotoxins

In addition to Thin-layer chromatography (TLC) methods, chromatographic methods, such as Liquid Chromatography (LC) and Gas Chromatography (GC) coupled to a specific detector, are the most commonly used techniques to date for obtaining highly accurate results. In the case of single mycotoxin analysis, e.g., AF, ochratoxin, the traditional LC with a FLD detector is the most widely used method for herbal medicine matrices. Currently, the co-occurrence of multiple mycotoxins has gained increasing attention. Therefore, liquid chromatography–tandem mass spectrometry (LC-MS-MS) is the technique choice for the simultaneous determination of various mycotoxins that belong to different chemical families.

#### 3.1.1. TLC Method

Since the discovery of AFs in 1961, TLC has been the traditional method used for the analysis of mycotoxins [165]. Along with the increased demand for data accuracy, separation and quantitation procedures have been gradually improved from TLC to HPLC. A review by Honma et al. [166] showed that the determinations of AFB_1_ in maize samples were mainly carried out using TLC methods in 1978. However, the percentage of TLC methods usage has been shown to decrease to 48% in 1989, and to 7% in 2002.

Currently, TLC methods are still recommended for the detection of AF in any plant material in the USP [47]. Due to a low detection cost and less demand on equipment, TLC methods are sometimes applied for the screening of mycotoxins in raw herbal drug materials when fungal analysis and related mycotoxin contamination are explored. Some examples are presented in Table 5.

#### 3.1.2. LC Technique

High-performance liquid chromatography (HPLC) with a FLD detector is perhaps the most commonly and widely used approach for AF determination in herbal medicine matrices, and has been recommended in pharmacopeia in numerous countries and regions [44,47,50,51,52]. AFB_1_ and AFG_1_ fluorescence is significantly quenched using aqueous solvent mixtures in reverse-phase chromatography. Therefore a derivatization reaction is typically carried out for determination. Over the past fifteen years, both pre- and post-column derivatization protocols have been proposed.

In the case of pre-column derivatization, the sample should be derived prior to the detection procedure. Trifluoroacetic acid (TFA) is the commonly used derivatization regent [66,75,76,107,108,109,110,111,112]. In general, the pre-column derivatization process involves a complex and time-consuming concentration procedure that cannot be performed by an on-line operation.

Compared to pre-column derivatization, post-column derivatization was reported more. Three types of post-column derivatization methods have been proposed including chemical, photochemical, and electrochemical derivatization methods. For chemical derivatization, iodine and pyridinium hydrobromide perbromide are typically used as derivatization reagents, and an additional pump and heating system is typically used [95,96,97,98,99,100,101,102,103]. Therefore, the post-column electrochemical and photochemical derivatization methods have obvious advantages, due to the fact that the operating procedures are easier to carry out and that they provide a higher sensitivity and wider linearity range [66,90,102].

Due to the complicated chemical constitution in herbal medicines, interference from sample matrices may be encountered at the retention times of analytes, resulting in incorrect identification of analyte. As a rule, these results require further confirmation using more reliable detectors, such as mass spectrometry, which is most commonly used in studies. However, some studies have also proposed an alternative strategy for the analyte confirmation. This strategy involves comparing HPLC chromatograms of sample extract with derivatization and underivatization for the fluorescence intensities of AFB_1_ and AFG_1_. In addition, they propose to perform re-measurements by adjusting the polarity of the mobile phase in order to overcome interference problem [88,93,107]. This technique provides a practical approach for laboratories without an expensive mass spectrometer.

The high-performance liquid chromatography with fluorescence detection (HPLC-FLD) method has also been proposed to use for the determination of OTA in herbal medicines [76,80,81,93,114]. In addition, the simultaneous determination of AFs and OTA using HPLC-FLD was recently developed [83,86,87]. This is likely attributed to the successful application of AlflaOchra Test^TM^ immunoaffinity columns in the herbal medicine matrix pretreatment, allowing for the simultaneous purification of AF and OTA.

While less investigated compared to AF and OTA, the determination of some other mycotoxins in herbal medicine matrices using HPLC-FLD was proposed, including CIT [72,117,118], ZEN [119], and FBs [101].

Recently, ultra-high performance liquid chromatography (UPLC) was used to analyze mycotoxins in herbal medicines [113,115,131]. Compared to traditional HPLC, UPLC was demonstrated to significantly improve chromatographic resolution and sensitivity and reduce the analysis cycle. These properties are suitable for high-throughput detection of trace complex mixtures. The technological progress of UPLC deserves mention: In 2012, Waters launched and trademarked the Waters^®^ ACQUITY^®^ UPLC^®^ Fluorescence (FLR) Detector with a large volume flow cell. It can be used to detect AFs without derivatization. Wen et al. [113] combined UPLC with FLR in order to develop a method for the simultaneous analysis of AFs and OTA in ginger and other related products. Compared to their prior work using HPLC-FLD and post-column photochemical derivatization [86], a comparable AFB_1_ and AFG_1_ sensitivity and an obviously increased AFB_2_ and AFG_2_ sensitivity were achieved in only half of the measurement time.

In addition to FLD and MS, other detectors have also been combined with HPLC for the detection of mycotoxins in herbal medicines, including UV and ELSD. Yue et al. [71] developed a method for the simultaneous determination of DON and NIV in TCMs using HPLC-UV. While the sensitivity of this method was found to be decreased relative to that obtained using GC-ECD [179], the sample pretreatment protocol was more efficient for routine analysis, as it did not include a derivatization step. The work of Wu et al. [120] demonstrated that their HPLC-ELSD method provided a convenient and reliable alternative to commonly used HPLC-FLD methods for the rapid determination of ZEN content, as it used a relatively simple QuEChERS method for sample cleanup.

As depicted in Table 3, the method based on HPLC/UPLC with FLD or UV for mycotoxin analysis in herbal medicine matrices typically involves a sample pretreatment step with sufficient selectivity such as IAC. This greatly narrows its application, as IACs are not available for all mycotoxin types.

Along with the popularization of modern MS technology, LC-MS/MS has been used increasingly for the quantitative analysis of mycotoxins in herbal medicine matrices in recent years. As depicted in Table 4, an upward trend is seen to be developing for the method used for the simultaneous determination of mycotoxins with great chemical diversity. This is something that is not achievable using HPLC with fluorescent or UV detection. Currently, it has been demonstrated that as many as 35 different toxins can be detected within one run by LC-MS/MS in herbal medicine matrices [13].

However, when using electrospray ionization, the ME will present a significant challenge that will affect the quantitative accuracy of determination by LC-MS/MS in complicated samples [180]. Primarily, different analytes vary with MEs, and substantial variations in signal suppression/enhancement exist between different sample matrices [13,146]. This leads to a challenging compensation. It is assumed that the use of an external matrix-matched calibration or an internal standard (IS) can function to minimize variations between samples. Stable isotopically labeled internal standards (SILIS) have been demonstrated to provide significant benefits for the correction of signal deviation, as they possess very similar chemical properties and identical retention times as the non-labeled substances [181]. However, SILIS are not commercially available for all mycotoxins, and many of these SILIS are very expensive. Therefore, reports involving SILIS are only aimed towards the analysis of a single type of mycotoxin [32,69,129]. Currently, due to easily operating, external matrix-matched calibration is commonly used to circumvent MEs for the simultaneous determination of multiple mycotoxins [74,78,135,137,138,141,142,145,146,147,148]. In order to ensure reliable quantitation, some studies combine ISs and the matrix calibration in order to eliminate MEs [13,70,139,143]. In some conditions, standard addition was carried out in order to correct quantitative analysis when matrix-matched calibration was unable to be applied due to matrix differences between different samples [12].

#### 3.1.3. GC Technique

GC was used as an alternate method for mycotoxin determination, especially when MS detectors are widely used. This allows for simultaneous qualitative and quantitative measurements of single or multiple analytes. An additional derivatization step, such as silylation or acylation, is commonly required following sample cleanup treatment. This is due to the fact that some mycotoxins are not sufficiently volatile at the column temperature, or cannot be converted into volatile derivatives [154,182,183].

As summarized by Lattanzio et al. [184] and Meneely et al. [154], there were numerous studies regarding the application of GC or GC-based methods for the determination of trichothecenes in food and feed samples. However, only few studies have addressed herbal medicine matrices. In 2009, the method for determination of T-2 toxin in traditional Chinese herbal medicines using GC-ECD was developed by Yue et al. [185]. In order to improve the selectivity and sensitivity of this method, an immunoaffinity column was used to purify the analyte in place of gel permeation chromatography filled with Bio-Beads S-X3 and Mycosep 225 column, which were used for the cleanup protocol in earlier studies [186,187]. Derivatization was carried out using 50 μL 25% (*v*/*v*) *n*-(heptafluoro-n-butyl) imidazole (HFBI) solution at 60 °C for 1 h. The derivatized heptofluorobuturyl ester was then extracted using 50 μL *n*-hexane and 200 μL aqueous sodium bicarbonate (4%) for determination. The limit of detection (LOD) of this method was determined to be 2.5 μg kg^−1^. This LOD was significantly lower than the LOD of 100 μg kg^−1^ achieved in an earlier study regarding T-2 detection in grain matrix [186]. In 2010, a similar sample pretreatment and derivatization protocol was successfully applied for DON detection in medicinal herbs and related products using the GC-ECD method [179]. The LOD and limit of quantification (LOQ) were determined to be 2.0 μg kg^−1^ and 5.0 μg kg^−1^, respectively, which met the most rigid European regulatory standard (200 μg kg^−1^). This method was applied for the analysis of real samples. Positive samples with DON contamination levels were found to range from 17.2 to 50.5 μg kg^−1^, which was further confirmed using GC-MS. In 2012, Kong et al. [188] further extended this method for the simultaneous determination of T-2 and HT-2 toxins. Following thorough validation, this was used for the analysis of TCMs and related products from various sources.

While high sensitivity was achieved, no significant advances were reported in relation to GC or GC-based methods for the analysis of mycotoxins in herbal medicine matrices in the past five years. This is likely attributed to the increasing application of LC-MS technology, which is more efficient in regards to time and can be carried out in the absence of complex derivatization procedure.

### 3.2. Rapid Screening Technologies for Mycotoxin Analysis

Rapid screening assays are critical tools for monitoring mycotoxin levels in herbal medicines. Most immunological techniques can be utilized for rapid screening. In general, these techniques are qualitative assays that demonstrate either the presence or absence of mycotoxins in herbal medicines. Screening assays have superior properties, including the speed at which the analysis is carried out, the simplicity of sample preparation, and the low cost per analysis. However, these methods also possess drawbacks as well, with the most notable one being reliability, as false-positive results are often obtained. However, different types of immunological techniques are continuing to emerge and are being rapidly developed.

#### 3.2.1. Enzyme-Linked Immunosorbent Assay (ELISA)

ELISA was first applied in the early 1970s and is a detection assay that combines specific immune response between an antigen and an antibody with efficient catalytic enzymes [189]. Currently, ELISA is likely the most commonly used antibody-based assay due to its advantages, including high specificity, rapid speed, simple design, and relatively inexpensive equipment. Some commercial ELISA test kits are available for common mycotoxins, including AFs, OTA, trichothecenes, FBs, and CIT [190]. However, the majority of these test kits are geared towards the food or feed matrix. Therefore, some studies have indicated that the complicated co-extract of herbal samples, especially the analogs of analytes, could lead to non-specific reactions of antibodies, leading to an overestimate of contaminants at very low concentrations [191,192,193]. In an effort to overcome the ME, matrix-matched calibration was used in some studies [193,194]. Other studies have proposed purification of sample extract using a multifunctional column or IAC prior to the ELISA test [195,196]. Recently, it has been proposed that ELISA should be used only as a qualitative preliminary screen, and samples which are test positive by ELISA should be further confirmed and exact contaminate levels should be quantified using LC-MS/MS or HPLC [191,197,198,199,200]. The Summary of some publications regarding mycotoxin determination in herbal matrices by ELISA is compiled in Table 6.

#### 3.2.2. Lateral Flow Immunoassay (LFIA)

Since its initial development in the 1980s, immunochromatographic test strip, also known as LFIA, has gained acceptance within the field across the world [205]. This method is an efficient screening technology that holds great advantages, including rapidity and simplicity, and relies on the transport of a particulate conjugate tag antibody (or antigen) probe to a specific antigen (or antibody) immobilized on the surface of a porous membrane [206]. As highlighted in a review published in 2013, LFIA has been used to rapidly screen for the presence of a single mycotoxin such as AF, OTA, AFM_1_, FBs, DON and ZEN in a variety of food or feed commodities [207]. In addition, multi-component immunochromatographic assays are available for the simultaneous determination of AFB_1_, OTA and ZEN in agro-food [208]. In a study carried out in 2015 [209], a colloidal gold test strip base on LFIA technique was developed for the detection of AFB_1_ in lotus seeds. This is a commonly used edible herbal medicine, and is particularly vulnerable to AF contamination. This proposed method involved a simple sample pretreatment step using 5 g of each sample extracted with 10 mL methanol-water (80:20, *v*/*v*) and diluted four folds with PBS (pH 7.4). The LOD of the test strip was determined to be 2.5 ng mL^−1^. Following confirmation by LC-MS/MS, no false positive or negative results were found. However, there have been no significant advances for the application of LFIA in herbal medicines over the recent years. The main reason for this limitation could have to do with how to avoid interference from the complicated matrix for the determination of trace analytes. Studies have proposed approaches aimed to minimize the ME, as summarized by Xie et al. [210]. These approaches include avoiding the use of organic solvents in the extraction procedure, using lower organic solvent concentrations in the extraction solution and dilution step, and applying a moderate volume of extraction solution with gentle stirring for a shorter time instead of conventional extraction method that requires robust shaking with high volumes of liquids for long periods of time.

#### 3.2.3. Aptamer-Based Lateral Flow Assay

**Figure 2:**
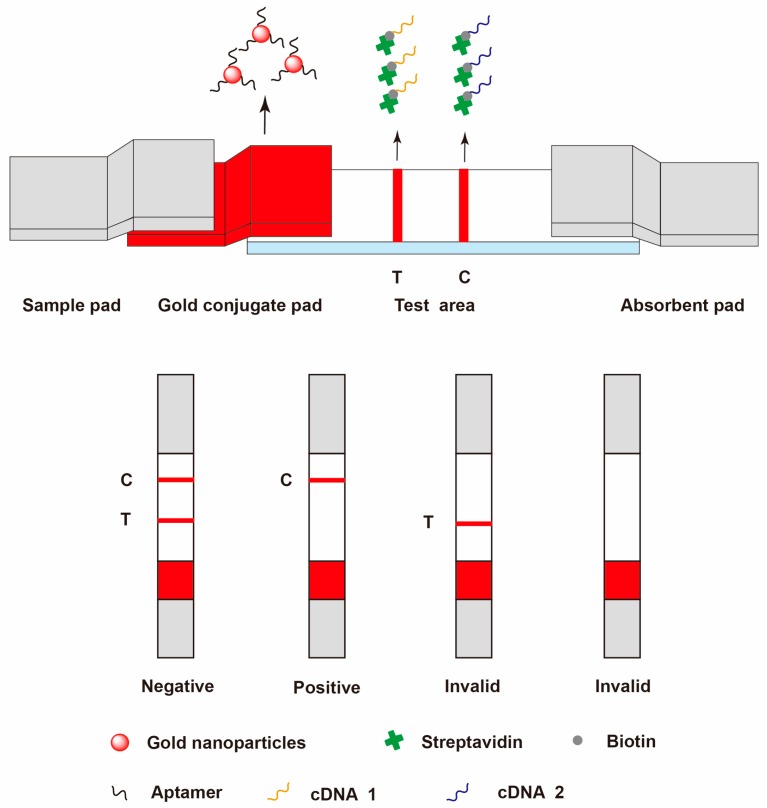
Schematic illustration of aptamer-based lateral flow strip for detection of OTA [213].

The aptamer-based lateral flow assay is an attractive method due to its inherent advantages of using aptamers compared to antibodies. Commonly, nucleic acid-based aptamers exhibit greater resistance to rough chemical conditions, including pH, ion strength, and organic solvent. This enables aptamers to better retain high sensitivity and specificity. In this respect, aptamers are preferable for use in complicated matrices. Aptamer-based lateral flow assays have been developed for the rapid screening of OTA in red wine [211] and for AFB_1_ in corn sample [212]. Recently, an aptamer-based lateral flow strip was proposed for the on-site detection of OTA in *Astragalus membranaceus*, a frequently used TCM [213] (Figure 2). In the test protocol, one gram of a test sample is simply extracted using 2.5 mL of methanol-water (80:20, *v*/*v*) and diluted four folds with a working buffer in order to eliminate interference from the matrix and methanol. Following optimization of critical testing parameters, a visual LOD of 1 ng mL^−1^ was obtained with higher specificity, and the screening procedure could be carried out in 15 min. A total of one of nine samples was found to be positive for OTA, and these results were in agreement with LC-MS/MS analysis. We note that the proposed method was only applied towards *Astragalus membranaceus* matrix, and the suitability of other matrices should be further studied.

#### 3.2.4. Cytometric bead array (CBA)

**Figure 3:**
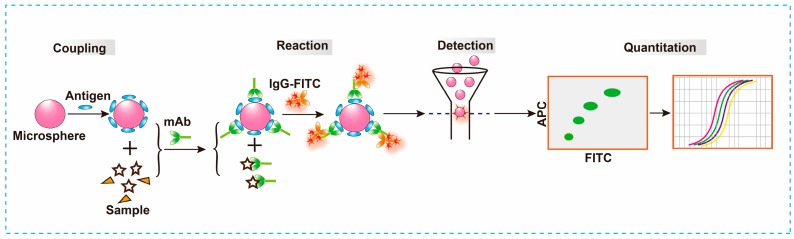
Schematic illustration of the fluorescent microspheres-based CBA assay for the detection of OTA [220].

CBA is a suspension array based on optically encoded microbeads. This technology is rapidly expanding for detection in both basic biological research and clinical diagnostic applications. CBA can provide sensitive, rapid, and real-time analysis for trace small molecules such as mycotoxins and pesticides. In addition, CBA is particularly suitable for on-site high-throughput detection. Recently, CBA has been used for both single [214,215,216] and multiple mycotoxin [217,218,219] detection in food and feed. In 2016, Xiao et al. [220] developed a cytometric bead array (CBA) method based on the indirect competition principle for the detection of OTA in Malt (Figure 3), a common Chinese herbal medicine. This is the first time that CBA technology was utilized for mycotoxin analysis in herbal medicines. Specifically, one gram of sample was extracted using 2 mL of 60% methanol and diluted 5 times with 20% methanol-PBS. Next, the OTA presented in the sample extract was competed with the BSA-OTA conjugated on the surface of fluorescence-encoded microsphere in order to attach to the anti-OTA monoclonal antibody (mAb). Then, FITC-IgG was bound to the captured antibody on the microsphere, and an accurate OTA quantitation was carried out using FITC fluorescence intensity measurements on the microsphere surface. Following optimization of critical parameters, the LOD of the method was determined to be 0.12 ng mL^−1^, which was 1.2 μg kg^−1^ multiplied by the dilution factor. A total of two of the sixteen samples were determined as positive samples, with contaminate values of 3.36 and 3.83 μg kg^−1^. These results were further confirmed by LC-MS/MS in order to prevent false-positive results. The method was recently modified by the same group based on the original basis [221]. This modification included using magnetic microspheres instead of polystyrene microsphere in order to simplify the separation and wash steps.

However, CBA has not yet been used extensively as a tool for routine mycotoxin analysis, due to the high cost and the complexity of the instruments required.

## 4. Conclusions

Herbal medicines have been used for disease prevention and treatment worldwide. Mycotoxin contamination represents one of the most critical toxicities present in herbal medicine and has been a concern globally. The effective control of mycotoxin contamination requires accurate and sensitive analytical methods.

Sample pretreatment has always been a challenging step for trace analysis in herbal medicine matrices. Because of the sophisticated and various compositions of herbs, especially in the case when multiclass mycotoxins are simultaneously determined, sample preparation protocols must often be optimized in order to increase the extraction efficiencies. Significant advances have been made in the cleanup protocol with the emergence of traditional IAC and SPE. Novel nanomaterials have been applied as absorbents, which were demonstrated to improve specificity relative to conventional types. In addition, AAC and MIP columns were introduced as an alternative to IAC, as they can be recycled in order to reduce costs.

Chromatographic techniques play a critical role in the analysis of mycotoxins in herbal medicine matrices. AFs, which are regulated by various pharmacopeias, are commonly identified with HPLC-FLD using different derivatization methods. However, in recent years, the co-occurrence of multiple mycotoxins in herbal medicines has drawn great attention within the field. Developments based on a modified QuEChERS procedure or multiple functional column purification combined with LC-MS/MS has functioned to increase the scope of mycotoxins that can be simultaneously analyzed.

While some analytical methods have been proposed, most are only aimed towards a single matrix. Thus, a more universal approach should be developed in order to extend the application scope, such as classifying the analytical method based on the basis of matrix variations.

Another issue in mycotoxin determination is the development of a rapid detection method. Conventional analytical methods always involve a more complex sample pretreatment protocol and a long detection time. These parameters limit their applications in high-throughput screening methods for mycotoxins. Thus, an increased interest has developed in the generation of a rapid detection approach. However, while there have been several types of rapid methods that have been successfully used, these have only been used for single herbal medicine matrices. Therefore, the challenge that exists for an analytical chemist is to broaden the application scope of the current methods, and work to develop a new rapid system that can use a variety of formats and platforms that are suitable for mycotoxin detection in herbal medicines.

Although a number of emerging mycotoxins have been investigated in herbal medicine, little attention has paid to their conjugates, which can escape conventional analytical detection of parent (free) forms. In the future, it is recommended the related research be extended in herbal medicine samples. For example, estimating the potential risk of some samples that are commonly used and particularly vulnerable to mycotoxin contamination (e.g., coix seed, malt, medicated leaven), as well as performing novel types of conjugated mycotoxin screening and preparing their reference substances.

Finally, mycotoxin analysis in herbal medicine would greatly benefit from the development of novel and effective sample extraction technologies. In addition, advances must be made in screening assays to enable on-site detection for mycotoxin analysis in herbal medicine.

## Figures and Tables

**Table 1:** Typical mycotxoins investigated in herbal medicines.

Type of Mycotoxin	Source and Solubility	References	
		Source	Solubility
Aflatoxin (AFB_1_, AFB_2_, AFG_1_, AFG_2_, AFM_1_)	**Main source:** *Aspergillus* **Solubility:** soluble in moderately polar organic solvents (e.g., chloroform, methanol, dimethysulfoxide), scarcely soluble in water (10–30 mg/mL) and insoluble in non-polar organic solvents	[16,17,18]	[18]
Ochratoxins (OTA, OTB)	**Main source:** *Aspergillus* and *Penicillium* **Solubility:** OTA: moderately soluble in polar organic solvents (e.g., chloroform, methanol) and dissolves in dilute aqueous sodium bicarbonate	[16,17,18]	[18]
Trichothecenes (Type A trichothecenes: (T-2, HT-2, NEO, DAS), Type B trichothecenes (DON, NIV, DOM-1, Fusarenone-X))	**Main source:** *Fusarium*, *Myrothecium*, *Stachybotrys*, *Trichoderma*, *Cephalosporium*, *Trichothecium* and *Verticimonosporium* **Solubility:** Type A trichothecenes: highly soluble in ethyl acetate, acetone, chloroform, dichloromethane and diethyl ether; Type B trichothecenes: soluble in methanol, acetonitrile and ethanol	[18,19]	[18]
Zearalenones (ZEN, α-ZOL, β-ZOL, ZAN)	**Main source:** *Fusarium* **Solubility:** ZEN: soluble in water, slightly soluble in hexane and progressively more soluble in benzene, acetonitrile, dichloromethane, methanol, ethanol and acetone	[16,18]	[18]
Fumonisins (FB_1_, FB_2_, FB_3_)	**Main source:** *Fusarium* **Solubility:** soluble in water, acetonitrile–water or methanol, and insoluble in chloroform and hexane	[16,17,18]	[18]
Alternaria toxins (AOH, AME, TEA, TEN)	**Main source:** *Alternaria* **Solubility:** AME: insoluble in aqueous NaHCO_3_ or water, slightly soluble in ether, sparingly soluble in benzene AOH: insoluble in hexane, light petroleum, benzene, aqueous NaHCO_3_ and water, more soluble than AME in ethanol, methanol, acetone TEA: slightly soluble in water TEN: slightly soluble in benzene	[10,20]	[21]
Patulin	**Main source:** *Penicillium* **Solubility:** soluble in water, methanol, ethanol, acetone and ethyl or amyl acetate and less soluble in diethyl ether and benzene	[18]	[18]
Citrinin	**Main source:** *Aspergillus*, *Penicillium* and related species **Solubility:** practically insoluble in water, soluble in ethanol, dioxane, dilute alkali, acetone, benzene, and chloroform	[18]	[22]
Cyclopiazonic acid	**Main source:** *Penicillium* and other fungi species including *Aspergillus* **Solubility:** soluble in chloroform and dimethyl sulfoxide	[18]	[18]
Sterigmatocystin	**Main source:** *Aspergillus* **Solubility:** highly soluble in pyridine	[18]	[23]
Gliotoxin	**Main source:** a wide variety of widespread moulds **Solubility:** soluble in pyridine, dioxane, dimethylformamide, acetic acid, and chloroform, slightly soluble in benzene, acetone, carbonate trachloride, and ethyl alcohol	[18]	[24]
Tremorgenic mycotoxins (Penitrem A, Verruculogen)	**Main source:** a wide spectrum of fungi belonging to the genera *Penicillium*, *Aspergillus*, *Claviceps* and *Acremonium* **Solubility:** Penitrem A: soluble in acetone, methanol and dimethyl sulfoxide. Verruculogen: soluble in benzene, ethyl acetate, and acetone, slightly soluble in ethanol, and very soluble in chloroform	[18]	[25,26]
Penicillic acid	**Main source:** several species of *Aspergillus* and *Penicillium* **Solubility:** moderately soluble (2%) in cold water and in cold benzene, highly soluble in hot water, alcohol, ether, and chloroform, and insoluble in pentane-hexane	[18]	[27]
Chaetoglobosin A	**Main source:** *Chaetomium globosum* and some species of *Penicillium* **Solubility:** soluble in acetone, methanol	[28,29,30]	[28,29]
Beauvericin and other enniatins (BEA, ENN A, ENN A_1_, ENN B, ENN B_1_)	**Main source:** *Fusarium* **Solubility:** having low solubility in water	[31]	[31,32]
Moniliformin	**Main source:** *Fusarium* **Solubility:** soluble in water and polar solvents	[18]	[18]

AFB_1_: Aflatoxin B_1_, AFB_2_: Aflatoxin B_2_, AFG_1_: Aflatoxin G_1_, AFG_2_: Aflatoxin G_2_, AFM_1_: Aflatoxin M_1_, OTA: Ochratoxin A, OTB: Ochratoxin B, T-2: T-2 toxin, HT-2: HT-2 toxin, NEO: Neosolaniol, DAS: Diacetoxyscirpenol, DON: Deoxynivalenol, NIV: Nivalenol, DOM-1: Deepoxydeoxynivalenol, ZEN: Zearalenone, α-ZOL: α-Zearalenol, β-ZOL: β-Zearalenol, ZAN: Zearalanone, FB_1_: Fumonisin B_1_, FB_2_: Fumonisin B_2_, FB_3_: Fumonisin B_3_, AOH: Alternariol, AME: Alternariol-methyl ether, TEA: Tenuazonic acid, TEN: Tentoxin, BEA: Beauvericin, ENN A: Enniatins A, ENN A_1_: Enniatins A_1_, ENN B : Enniatins B, ENN B_1_: Enniatins B_1._

**Table 2:** Maximum recommended levels of AFs and OTA in medicinal plants.

Country/Region	Product (Group)	AFB_1_ μg kg^−1^	Total AFs μg kg^−1^	OTA μg kg^−1^	Reference
Europe ^a^	Herbal drugs	2	4		[44]
United States	Some types of raw medicinal herb materials, as well as their powder and/or dry extract	5	20		[47,48]
China	A total of nineteen different types of TCMs	5	10		[50]
Britain	Herbal drugs	2	4		[45]
Korea	Armeniacae Semen, Arecae Semen, Cassiae Semen, Crotonis Semen, Curcumae Radix, Dolichoris Semen, Glycyrrhizae Radix et Rhizoma, Nelumbinis Semen, Myristicae Semen, Persicae Semen, Pinelliae Tuber, Polygalae Radix, Carthami Flos, Thujae Semen, Trichosanthis Semen, Zizyphi Semen	10	15		[51]
Indonesia	Coconut, spices and traditional drug medicines/herbs		20		[42]
Canada	Products containing ginseng or any substance derived from this source, Evening Primrose Oil, sugar cane, sugar beets, cottonseed	5	20		[49]
Japan	Crude drug and preparations containing crude drugs as main ingredient (crude drug preparations)		10		[52]
Vietnam	Nutmeg	5	10	30	[53]
	Ginger and turmeric				
	Black and white pepper				
	Licorice root used for herbal tea			20	
	Licorice extract for beverage or to mix			80	
Germany	Any materials used in manufacture of medicinal products (including medicinal herbal products)	2	4		[46]
Argentina	Herbs, herbal materials and herbal preparations used for herbal tea infusions	5	20		[46]
Europe ^b^	Nutmeg	5	10	15	[43]
	Ginger				
	Turmeric				
	White and black pepper				
	Dried figs	6	10		
	Liquorice root, ingredient for herbal infusion			20	
	Liquorice extract, for use in food in particular beverages and confectionary			80	

^a^ Recommended by European Pharmacopoeia Commission; ^b^ Recommended by European Commission.

**Table 3:** Overview on HPLC methods in mycotoxins analysis in herbal medicines.

Mycotoxin	Detection	Sample	Extraction Solution	Extraction Method	Cleanup	LOD	LOQ	Reference
AFs	HPLC-FLD post-column Photochemical derivatization	Ginseng, ginger	Methanol-10 mM PBS containing 1% Tween 20 (80:20, *v*/*v*)	Shaking	IAC	0.1 ng g^−1^ for AFB_1_	1 ng g^−1^ for AFB_1_	[80]
AFs	HPLC-FLD post-column Photochemical derivatization	Ginger	Methanol-0.5% NaHCO_3_ solution (70:30, *v*/*v*)	Shaking	IAC			[81]
AFs	HPLC-FLD post-column Photochemical derivatization	Ginseng, ginger, kava kava, black cohosh, echinacea, valerian	Acetonitrile–water (84:16, *v*/*v*)	Shaking	IAC			[82]
AFs	HPLC-FLD post-column Photochemical derivatization	*Glycyrrhiza uralensis*	Methanol-water (80:20, *v*/*v*)	Sonicating	IAC	0.015–0.06 μg kg^−1^	0.05–0.2 μg kg^−1^	[83]
AFs	HPLC-FLD post-column Photochemical derivatization	Nelumbinis semen	Methanol-water (80:20, *v*/*v*)	Homogenizing	IAC	0.03–0.10 μg kg^−1^	0.06–0.25 μg kg^−1^	[84]
AFs	HPLC-FLD post-column Photochemical derivatization	Traditional Chinese medicine Yinpian	Methanol-water (70:30, *v*/*v*)	Sonicating	IAC	0.12–0.44 pg	0.31–1.09 pg	[85]
AFs	HPLC-FLD post-column Photochemical derivatization	Ginger and related products	Methanol-water (80:20, *v*/*v*)	Sonicating	IAC	0.03–0.2 μg kg^−1^	0.1–0.6 μg kg^−1^	[86]
AFs	HPLC-FLD post-column Photochemical derivatization	Nutmeg	Methanol-water (80:20, *v*/*v*)	Sonicating	IAC	0.02–0.06 μg kg^−1^	0.06–0.2 μg kg^−1^	[87]
AFs	HPLC-FLD post-column Photochemical derivatization	Chinese herbal pieces	Methanol-water (70:30, *v*/*v*)	Sonicating	IAC			[88]
AFs	HPLC-FLD post-column Photochemical derivatization	Coix seed	Methanol-water (80:20, *v*/*v*)	Sonicating	IAC	0.01–0.11 μg kg^−1^	0.04–0.32 μg kg^−1^	[89]
AFs	HPLC-FLD post-column Photochemical derivatization	Fructus Bruceae	Methanol-water (80:20, *v*/*v*)	Sonicating	IAC	0.02–0.08 ng mL^−1^	0.05–0.20 ng mL^−1^	[90]
AFs	HPLC-FLD post-column Photochemical derivatization	Shujin Huoxue pills	Methanol-water (70:30, *v*/*v*)	Sonicating	IAC	0.26–1.04 pg		[91]
AFs	HPLC-FLD post-column Photochemical derivatization	Sterculiae Lychnophorae	Methanol-water (70:30, *v*/*v*)	Sonicating	IAC	0.0144–0.0528 μg L^−1^	0.0288–0.1056 μg L^−1^	[92]
AFs	HPLC-FLD post-column Photochemical derivatization	Spices	Methanol-water (70:30, *v*/*v*) or Methanol-water (80:20, *v*/*v*)	Shaking	IAC	0.01 ng g^−1^ for each AF		[93]
AFs	HPLC-FLD post-column Photochemical derivatization	Red pepper, black pepper, turmeric and cinnamon	Methanol-water (80:20, *v*/*v*)	Homogenizing	IAC	0.02–0.08 ng g^−1^		[38]
AFs	HPLC-FLD post-column Photochemical derivatization	Six kinds of medicinal herbs	Methanol-water (70:30, *v*/*v*)	Homogenizing	IAC	0.04–0.2 μg kg^−1^	0.25–1.0 μg kg^−1^	[94]
AFs	HPLC-FLD post-column bromination derivatization	Twelve kinds of spices	Methanol-water (80:20, *v*/*v*)		IAC	1 μg kg^−1^		[95]
AFs	HPLC-FLD post-column bromination derivatization	Thirty seven TCMs	Methanol-water (70:30, *v*/*v*)	Sonicating	IAC	0.06–0.20 μg kg^−1^		[96]
AFs	HPLC-FLD post-column bromination derivatization	Thirty three species of medicinal herbs and 11 kinds of patent medicines	Methanol-water (70:30, *v*/*v*)	Sonicating	IAC			[97]
AFs	HPLC-FLD post-column bromination derivatization	Herbal plants	Methanol-water		IAC	0.03–0.3 μg kg^−1^	0.05–0.7 μg kg^−1^	[98]
AFs	HPLC-FLD post-column iodine derivatization	Nighteen TCMs	Methanol-water (70:30, *v*/*v*)	Sonicating	IAC	0.22–0.75 μg kg^−1^		[99]
AFs	HPLC-FLD post-column iodine derivatization	Bulbus Fritillariae Thunbergii, Fructus Schisandrae Chinensis, Fructus Crataegi, Fructus Mume	Methanol-water (70:30, *v*/*v*)	Sonicating	IAC	0.06 μg kg^−1^	0.3 μg kg^−1^	[100]
AFs	HPLC-FLD post-column iodine derivatization	Sixteen plant species	Methanol-water (80:20, *v*/*v*)	Homogenizing	IAC	0.5 μg kg^−1^ for AFB_1_		[101]
AFs	HPLC-FLD post-column iodine derivatization	Citri Reticulatae Pericarpium	Methanol-water (70:30, *v*/*v*)	Shaking	IAC	0.19–0.24 μg kg^−1^		[102]
AFs	HPLC-FLD post-column iodine derivatization	Proprietary Chinese medicines	Methanol	Sonicating	GO-based dSPE	0.020–0.041 ng mL^−1^	0.061–0.125 ng mL^−1^	[103]
AFs	HPLC-FLD post-column derivatization with electrochemically generated bromine	Twenty-eight samples of herbal medicinal products	Methanol-water (80:20, *v*/*v*)	Homogenizing	IAC	0.04 ng g^−1^		[104]
AFs	HPLC-FLD post-column derivatization with electrochemically generated bromine	Five kinds of medicinal herbs	Methanol-water (70:30, *v*/*v*)	Sonicating	IAC		0.05–0.1 ng g^−1^	[105]
AFs	HPLC-FLD post-column derivatization with electrochemically generated bromine	One hundred and three samples of different kinds of spices and herbs	Methanol-water (80:20, *v*/*v*)	Shaking	IAC	0.2–0.5 μg kg^−1^	0.6–1.5 μg kg^−1^	[67]
AFs	HPLC-FLD post-column derivatization with electrochemically generated bromine	Dried figs	Methanol-water (80:20, *v*/*v*)		IAC	0.1 ng g^−1^		[106]
AFs	HPLC-FLD post-column derivatization with electrochemically generated bromine	Citri Reticulatae Pericarpium	Methanol-water (70:30, *v*/*v*)	Shaking	IAC	0.10–0.18 μg kg^−1^		[102]
AFs	HPLC-FLD post-column derivatization with electrochemically generated bromine	One hundred and eighty five functional food and 56 herbal medicines	Methanol-water (70:30, *v*/*v*)	Shaking	IAC	0.07–0.32 ng g^−1^	0.21–0.96 ng g^−1^	[66]
AFs	HPLC-FLD pre-column derivatization with TFA	White pepper	Chloroform-water (100:10, *v*/*v*)		Silica cartridge and C_18_ cartridge	0.006–0.009 μg L^−1^		[75]
AFs	HPLC-FLD pre-column derivatization with TFA	Ninety one spice samples	Methanol-water (80:20, *v*/*v*)	Shaking	IAC	0.1–0.2 μg kg^−1^		[76]
AFs	HPLC-FLD pre-column derivatization with TFA	Twenty three commercial traditional herbal medicines	Methanol-water (70:30, *v*/*v*)	Shaking	IAC	0.01 μg kg^−1^		[107]
AFs	HPLC-FLD pre-column derivatization with TFA	Ginseng roots	Methanol-water (80:20, *v*/*v*)	Shaking	IAC	0.1 ng g^−1^ for AFB_1_		[108]
AFs	HPLC-FLD pre-column derivatization with TFA	Eighty eight spices and processed spice products	Methanol-water (70:30, *v*/*v*)	Shaking	IAC	0.01–0.15 μg kg^−1^	0.03–0.45 μg kg^−1^	[109]
AFs	HPLC-FLD pre-column derivatization with TFA	Eight kinds of medicinal herbs	Methanol-water (80:20, *v*/*v*)	Blending	IAC	0.02–0.09 ppb		[110]
AFs	HPLC-FLD pre-column derivatization with TFA	Pu-erh tea	Acetonitrile-water (84:16, *v*/*v*)	Shaking	SPE			[111]
AFs	HPLC-FLD pre-column derivatization with TFA	One hundred and eighty five functional food and 56 herbal medicines	Methanol-water (70:30, *v*/*v*)	Shaking	IAC	0.32–2.28 ng g^−1^	0.95–6.83 ng g^−1^	[66]
AFs	HPLC-FLD pre-column derivatization with TFA	Black, White and Green Peppers	Acetonitrile-water (60:40, *v*/*v*)	Blending	IAC	0.01–0.5 ng mL^−1^	0.05–2.5 ng mL^−1^	[112]
AFs	HPLC-FLD	*Maytenus ilicifolia*	Methanol-water (70:30, *v*/*v*)	Sonicating	IAC	0.1–3.5 ng g^−1^		[68]
AFs	UPLC-FLD	Ginger and related products	Methanol-water (80:20, *v*/*v*)	Sonicating	IAC	0.005–0.2 μg kg^−1^	0.0125–0.5 μg kg^−1^	[113]
OTA	HPLC-FLD	Fifty-seven traditional Chinese medicinal plants	Methanol-water (80:20, *v*/*v*)	Sonicating	IAC	0.3 μg kg^−1^	0.8 μg kg^−1^	[114]
OTA	UPLC-FLD	Ginger	Acetonitrile–water (60:40, *v*/*v*)	Sonicating	MIP-SPE	0.09 ng mL^−1^	0.30 ng mL^−1^	[115]
OTA	UPLC-FLD	Ginger powder	Acetonitrile–water (60:40, *v*/*v*)	Sonicating	AAC	0.5 μg kg^−1^	1.5 μg kg^−1^	[116]
OTA	HPLC-FLD	Ginseng, Ginger	Methanol-1% NaHCO_3_ solution (70:30, *v*/*v*)	Shaking	IAC	0.1 ng g^−1^	1 ng g^−1^	[80]
OTA	HPLC-FLD	Ginger	Methanol-0.5% NaHCO_3_ solution (70:30, *v*/*v*)	Shaking	IAC			[81]
OTA	HPLC-FLD	*Glycyrrhiza uralensis*	Methanol-water (80:20, *v*/*v*)	Sonicating	IAC	0.25 μg kg^−1^	0.75 μg kg^−1^	[83]
OTA	HPLC-FLD	Ginger and related products	Methanol-water (80:20, *v*/*v*)	Sonicating	IAC	0.3 μg kg^−1^	0.9 μg kg^−1^	[86]
OTA	HPLC-FLD	Nutmeg	Methanol-water (80:20, *v*/*v*)	Sonicating	IAC	0.25 μg kg^−1^	0.8 μg kg^−1^	[87]
OTA	HPLC-FLD	Spices	Acetonitrile–water (60:40, *v*/*v*)	Shaking	IAC	0.10 ng g^−1^		[93]
OTA	HPLC-FLD	Black pepper, white pepper and spice mixture samples	1M phosphoric acid-chloroform (10:100, *v*/*v*)	Shaking	IAC	0.2 μg kg^−1^		[76]
OTA	UPLC-FLD	Ginger and related products	Methanol-water (80:20, *v*/*v*)	Sonicating	IAC	0.1 μg kg^−1^	0.3 μg kg^−1^	[113]
CIT	HPLC-FLD	Red mold rice	Ethanol-water (75:25, *v*/*v*)	Shaking				[72]
CIT	HPLC-FLD	Red yeast rice, medicinal plants and their related products	Methanol-water (70:30, *v*/*v*)	Vortexing	IAC	0.8 μg kg^−1^	2 μg kg^−1^	[117]
CIT	HPLC-FLD	Red fermented rice	Methanol-water (80:20, *v*/*v*)	Sonicating		0.0005 μg mL^−1^	0.001 μg mL^−1^	[118]
DON, NIV	HPLC-UV	Thirty samples of TCMs	Acetonitrile-water (80:20, *v*/*v*)	Homogenizing	SPE	63 μg kg^−1^ for DON and 50.0 μg kg^−1^ for NIV	125.0 μg kg^−1^ for DON and 100.0 μg kg^−1^ for NIV	[71]
ZEN	HPLC-FLD	One hundred and seven samples of Chinese medicinal herbs	Methanol-water (80:20, *v*/*v*)	Homogenizing	IAC	9.5 μg kg^−1^		[119]
ZEN	HPLC-ELSD	Barley	Methanol	Blending	QuEChERS	1.56 ng g^−1^		[120]
ZEN, α-ZOL, β-ZOL	HPLC-FLD	Coix seed	Methanol-water (80:20, *v*/*v*)	Sonicating	IAC	11.7–50.2 μg kg^−1^	29.3–125.5 μg kg^−1^	[89]
FB_1_, FB_2_, FB_3_	HPLC-FLD pre-column derivatization with o-phthaldialdehyde	Sixteen plant species	Methanol	Homogenizing	SPE	5 μg kg^−1^ for FB_1_		[101]

**Table 4:** Examples of analytical methods for mycotoxins analysis by LC-MS/MS in herbal medicine.

Mycotoxin	Sample	Extraction Solution	Extraction Method	Cleanup	LOD	LOQ	Reference
AFs	Zizyphi Fructus		SFE	Without purification	0.17–0.32 ng g^−1^	0.56–1.05 ng g^−1^	[121]
AFs	One hundred and seventy four samples from 50 medicinal herb species	Methanol-water (70:30, *v*/*v*)	Sonicating	IAC	0.135–0.883 μg kg^−1^		[122]
AFs	Lotus seeds	Methanol-water (80:20, *v*/*v*)	Blending	IAC	0.003–0.007 μg kg^−1^	0.010–0.020 μg kg^−1^	[123]
AFs	*Mucuna pruriens*, *Delphinium denudatum*, and *Portulaca oleraceae*	Methanol-water (70:30, *v*/*v*)	Sonicating	IAC	0.28–1.10 μg kg^−1^	0.79–3.34 μg kg^−1^	[124]
AFs	Armeniacae Semen Amarum	Acetonitrile-water (84:16, *v*/*v*)	Vortexing	Without purification	5.200–6.300 ng L^−1^	10.40–12.60 ng L^−1^	[125]
AFs	Walnut kernel	Methanol-water (70:30, *v*/*v*)	Sonicating	Self-made amino -function nanometre Fe_3_O_4_ magnetic polymer SPE	0.004–0.013 μg kg^−1^	0.012–0.042 μg kg^−1^	[126]
AFs	Twenty two TCMs matrix types	Methanol-water (70:30, *v*/*v*), Methanol-water (75:25, *v*/*v*), Methanol-water (85:15, *v*/*v*)	Sonicating, shaking, and homogenizing	C_18_-SPE	0.008–0.022 μg kg^−1^	0.011–0.029 μg kg^−1^	[79]
AFs, OTA	*Glycyrrhiza uralensis*	Methanol-water (80:20, *v*/*v*)	Sonicating	IAC	0.003–0.007 μg kg^−1^	0.010–0.020 μg kg^−1^	[127]
AFB_1_, OTA	Licorice roots, fritillary bulbs	Methanol-water (85:15, *v*/*v*)	Sonicating	C_18_-SPE	0.012 μg kg^−1^ for AFB_1_, 0.024 μg kg^−1^ for OTA	0.035 μg kg^−1^ for AFB_1_, 0.095 μg kg^−1^ for OTA	[77]
AFs, OTA, ST	Two-hundred and forty-four samples of 25 types of widely used TCMs	Acetonitrile–water (84:16, *v*/*v*)	Soaking and shaking		0.1–25.0 ng L^−1^		[128]
AFB_1_, AFB_2_, AFG_1_, AFG_2_, AFM_1_, AFM_2_	Thirty TCMs	Acetonitrile–water (84:16, *v*/*v*)	Homogenizing	Home-made mixed cartridge	0.07–0.26 μg kg^−1^	0.10–0.73 μg kg^−1^	[129]
AFs, PAT	Chinese patent medicines	Acetonitrile–water (84:16, *v*/*v*)	Vortexing	Mycosep 228 Aflapat mutifutional column	0.1–1 μg kg^−1^		[130]
OTA	Five types of TCMs	Acetonitrile–water (60:40, *v*/*v*)	Soaking	AAC	0.5–0.8 μg kg^−1^	1.5–2.5 μg kg^−1^	[131]
PAT	Fructus crataegi, fructus mume, pericarpium citri reticulatae, fructus aurantii	Pectinase enzymolysis and acetonitrile-water (60:20, *v*/*v*) extraction	Blending	dSPE and Mycrosep228AlaPat	0.3–0.5 μg kg^−1^		[132]
OTA, PAT	Seventy nine samples of various spices and herbs	Methanol-water (3:1, *v*/*v*) for FBs, acetonitrile–water (60:40, *v*/*v*) for OTA	Homogenizing for FBs, soaking for OTA	SAX cartridge for FBs, and IAC for OTA	0.1 ng g^−1^ for OTA, 0.5–1.0 ng g^−1^ for FBs		[133]
ZEN, α-ZOL	Twenty five TCMs	Methanol-water (80:20, *v*/*v*)	Shaking	IAC	0.6 μg kg^−1^	1.2 μg kg^−1^	[134]
ZEN, α-ZOL, β-ZOL, ZAN, α-ZAL, β-ZAL	Thirty-three commercially available dried TCMs	Acetonitrile–water (60:40, *v*/*v*)	Soaking and Homogenizing	Home-made cleanup cartridge	0.06–0.79 ng mL^−1^	0.13–0.99 ng mL^−1^	[70]
T2, HT-2, NEO, and DAS	Coix seed	Acetonitrile–water (84:16, *v*/*v*)	Sonicating	Magnetic SPE		0.3–1.5 μg kg^−1^	[135]
FB_1_, FB_2_ and FB_3_	Four types of dried TCMs	Acetonitrile–water (50:50, *v*/*v*)	Soaking and homogenizing	MultiSep 211 Fum columns	0.05–0.10 ng mL^−1^	0.08–0.16 ng mL^−1^	[69]
CIT	Twenty seven TCMs	Methanol-water (70:30, *v*/*v*)	Shaking	IAC	1.0 μg kg^−1^	2.5 μg kg^−1^	[136]
ENNs and BEA	Sixty types of dried Chinese medicinal herbs	Methanol	Shaking	Without purification	0.8–1.2 μg kg^−1^	2.5–3.7 μg kg^−1^	[32]
23 mycotoxins	Botanical food supplements	Ethyl acetate-formic acid (95:5, *v*/*v*)	Shaking	Oasis HLBTM SPE cartridges	0.3–30 ng g^−1^	1–100 ng g^−1^	[12]
22 mycotoxins	Raw tea and herbal infusion materials	Ethyl acetate-formic acid (99:1, *v*/*v*)	Shaking	NH_2_-SPE and C_18_-SPE column	2.1–122 μg kg^−1^	4.1–243 μg kg^−1^	[74]
35 mycotoxins	Four types of dried TCMs	Acetonitrile–water (84:16, *v*/*v*)	ASE	Homemade Cleanup Cartridges	0.01–1.56 μg kg^−1^	0.11–1.86 μg kg^−1^	[13]
15 mycotoxins	Milk thistle samples (seeds and extract)	30 mM NaH_2_PO_4_ buffer pH 7.1 and 5% formic acid in acetonitrile	Vortexing	QuEChERS	0.45–459 μg kg^−1^	1.5–1530 μg kg^−1^	[137]
17 mycotoxins	Puerariae lobatae radix	Acetonitrile–water (90:10, *v*/*v*)	Sonicating	PuriToxSR TC-M160 MultiPurification Column	0.00203–1.06 μg kg^−1^	0.0488–4.97 μg kg^−1^	[78]
10 mycotoxins	*Panax notoginseng*	Acetonitrile	Sonicating	HLB multifunction cleanup column	0.043–2.9 μg kg^−1^	0.15–8.6 μg kg^−1^	[138]
11 mycotoxins	*Morinda officinalis*	Methanol-water (80:20, *v*/*v*) containing 0.1% formic acid	Vortexing	Without purification	0.02–4.00 ng mL^−1^	0.06–10 ng mL^−1^	[139]
8 mycotoxins	*Angelica sinensis*	PBS and 5% formic acid in acetonitrile	vortexing	QuEChERS	0.005–0.125 μg kg^−1^	0.0625–0.25 μg kg^−1^	[140]
8 mycotoxins	Chinese yam and related products	Methanol-water-formic acid (79:20:1, *v*/*v*/*v*)	Sonicating	Without purification	0.02–0.15 ng mL^−1^	0.06–0.50 ng mL^−1^	[141]
21 mycotoxins	Radix Paeoniae Alba	PBS and 5% formic acid in acetonitrile	Vortexing	Modified QuEChERS	0.03–5.36 μg kg^−1^	0.20–22.50 μg kg^−1^	[142]
11 mycotoxins	*Areca catechu*	Methanol-water (80:20, *v*/*v*)	Soaking and vortexing	Without purification	0.1–20 μg kg^−1^	0.25–50 μg kg^−1^	[143]
11 mycotoxins	Malt	Acetonitrile-water-acetic acid (80:19:1, *v*/*v*/*v*)	Sonicating	Without purification	0.01–5.85 ng mL^−1^	0.03–17.5 ng mL^−1^	[144]
11 mycotoxins	Three types of ground herbs	Acetonitrile-water (50:50, *v*/*v*)	Shaking	A buffered QuEChERS SPE	0.5–4.0 μg kg^−1^	1.5–12 μg kg^−1^	[145]
11 mycotoxins	*Alpinia oxyphylla*	Acetonitrile -water-acetic acid (79:20:1, *v*/*v*/*v*).	Sonicating	Without purification	0.03–6.00 μg kg^−1^	0.10–20.0 μg kg^−1^	[146]
ZEN and type A trichothecenes	*Salviae Miltiorrhizae* Radix et Rhizoma	Acetonitrile-water (84:16, *v*/*v*)	Soaking and sonicating	Fe_3_O_4_/MWCNT	0.45–1.80 μg kg^−1^	1.20–4.80 μg kg^−1^	[147]

**Table 5:** Representative TLC methods for mycotoxin detection in herbal medicines.

Sample	Mycotoxin	Reference
A total of 152 samples, belonging to 56 species of medicinal herbs	AFB_1_, AFB_2_, AFG_1_, AFG_2_, ZEN, T-2, NEO, DON	[167]
Ninety-one samples of medicinal herbs, composed by 65 different plant species	AFB_1_, AFB_2_, AFG_1_, AFG_2_, OTA	[168]
A total of 30 raw materials comprising five samples of each medicinal	AFB_1_	[169]
A total of 68 powdered samples	AFB_1_, AFB_2_, AFG_1_, AFG_2_, CIT, ST	[170]
A total of 25 sun dried freshly stored fruit samples of and 25 powdered of *Emblica officinalis*, *Terminalia bellirica*, *Terminalia chebula*	AFB_1_, AFB_2_, AFG_1_, AFG_2_	[171]
Eighty samples consisting of 20 each of four medicinal plants	AFB_1_, AFB_2_, AFG_1_, AFG_2_	[172]
Two random samples of two different plant materials	AFB_1_, AFG_1_, CIT, Griseofulvin, OTA, ST	[173]
Thirty different samples of medicinal plants	AFB_1_, AFB_2_, AFG_1_, AFG_2_, OTA	[174]
Ten sun dried one year stored crude drug samples	AFB_1_, AFB_2_, AFG_1_, AFG_2_	[175]
A total of 210 samples randomly bought from traditional medical practitioners	AFB_1_, AFB_2_, AFG_1_, AFG_2_	[176]
A total of 63 samples which includes 38 different types of commonly used herbs, herbal products, spices, and food materials	AFB_1_, AFB_2_, AFG_1_, AFG_2_	[177]
Eighteen samples of 6 different types	AFB_1_	[178]

**Table 6:** Reported ELISA methods for mycotoxin detection in herbal medicines.

Sample	Mycotoxin	LOD	Reference
Red scaled, red and black pepper	AFs and AFB_1_	0.25 μg kg^−1^ for AFs, 1.0 μg kg^−1^ for AFB_1_	[199]
Black pepper, coriander, ginger and turmeric	OTA		[193]
*P. ginseng*, *P. quinquefolius*	ZEN		[200]
Eighty-four medicinal and/or aromatic herb samples	OTA, FBs, AFs, ZEN, T-2, DON, CIT	0.025 μg kg^−1^ for OTA, 83 μg kg^−1^ for FBs, 1.4 μg kg^−1^ for AFs, 0.14 μg kg^−1^ ZEN, 0.28 μg kg^−1^ for T-2, 14.80 μg kg^−1^ for DON and 16.5 μg kg^−1^ for CIT	[195]
A total of 700 herbal medicine samples (70 types and10 samples of each type)	AFB_1_	0.05 ng mL^−1^	[201]
Ninety three organic spice and 37 organic herb samples	AFB_1_		[196]
A total of 36 samples of spices	AFB_1_		[39]
Red chilli, black pepper, turmeric, coriander, cumin, fennel, caraway, fenugreek, and dry ginger	AFs, OTA, CIT	4 ng g^−1^ for AFs, 2 ng g^−1^ for OTA, and 15 ng g^−1^ for CTN	[197]
Lichens	AOL, AFB_1_, DON, DAS, ZEN, Mycophenolic acid (MPA), OTA, PR toxin (PR), ST, T2, FB_1_, Cyclopiazonic acid (CPA), CIT, Emodin (EMO), EA, Roridin A (ROA)	2 (AFB_1_, T2, EA), 4 (ST), 8 (OTA, ROA), 20 (MPA, CIT, AOL, ZEN), 40 (DON, EMO), 50 (FB_1_) and 100 (DAS, CPA, PR) ng g^−1^	[202]
Lotus seeds	AFB_1_	0.128 μg L^−1^	[194]
Cassava flour	AFs		[203]
Garlic	FB_1_, FB_2_	0.17 ppm	[198]
Ginger, galangal, garlic, elephant garlic	AFB_1_		[204]
